# High Burden of Occult Hepatitis B Infection in HIV-Infected Patients in Southern Vietnam: Insights from Serological and Molecular Analysis

**DOI:** 10.3390/ijms27157040

**Published:** 2026-08-05

**Authors:** Huynh Hoang Khanh Thu, Yulia V. Ostankova, Alexander N. Shchemelev, Elena N. Serikova, Vladimir S. Davydenko, Nadezhda A. Pechnikova, Tran Ton, Truong Thi Xuan Lien, Edward S. Ramsay, Areg A. Totolian

**Affiliations:** 1 Pasteur Institute in Ho Chi Minh City, Ho Chi Minh City 700000, Vietnam; thuhhk@pasteurhcm.edu.vn (H.H.K.T.); tont@pasteurhcm.edu.vn (T.T.); 2 Saint Petersburg Pasteur Institute, St. Petersburg 197101, Russia; shenna1@yandex.ru (Y.V.O.); genista.bio@gmail.com (E.N.S.); vladimir_david@mail.ru (V.S.D.); nikanobelevka@gmail.com (N.A.P.); totolian@spbraaci.ru (A.A.T.); 3 Faculty of Pharmacy, Van Lang University, Ho Chi Minh City 700000, Vietnam; lien.ttx@vlu.edu.vn

**Keywords:** hepatitis B virus (HBV), chronic HBV infection (CHB), occult HBV infection (OBI), escape mutations, people living with HIV (PLWH), Vietnam

## Abstract

Hepatitis B virus (HBV) infection remains a major concern among people living with HIV (PLWH), particularly in high-endemic settings such as Vietnam. The study evaluated the serological and molecular characteristics of HBV among PLWH receiving antiretroviral therapy (ART) in southern Vietnam. A cross-sectional study was conducted among 316 HIV-infected patients receiving ART. Serological markers (HBsAg, anti-HBs IgG, and anti-HBc IgG) and HBV DNA were assessed using highly sensitive assays, and the *Pre-S1/Pre-S2/S* regions were sequenced for genotype and mutation analysis. Associations were evaluated using appropriate statistical methods. HBV DNA was detected in 32.6% of the patients, whereas HBsAg was present in only 16.1%. The most common serological profile was susceptibility (36.4%), followed by occult HBV infection (OBI) (17.4%), including 6.6% with completely seronegative OBI, and chronic HBV (CHB) (15.8%). Males and older individuals showed a significantly higher risk of CHB (adjusted odds ratio [aOR] = 2.58 and aOR = 1.87, respectively). Genotype B predominated (78.4%) overall, but genotype C was significantly more frequent in OBI than in CHB patients (31.5% vs. 8.3%, *p* = 0.008). Moreover, the burden of surface *S* gene escape mutations was significantly higher in the OBI group (*p* = 0.023). The LASSO model showed a moderate discriminatory ability for predicting OBI status (Area Under the Receiver Operating Characteristic [ROC] Curve [AUC] = 0.76). Lamivudine-associated resistance (rtM204I/V) was the most common RT mutation detected in the overlapping *RT/S* region. HIV-infected individuals in southern Vietnam face a burden of both CHB and OBI, including a high proportion of seronegative OBI. The presence of seronegative OBI further supports the risk of underdiagnosis when relying only on standard serological markers, and reflects the need for integrated strategies combining highly sensitive serological assays with molecular diagnostics to improve HBV detection among high-risk populations.

## 1. Introduction

Bloodborne pathogens remain a significant global public health concern, in which hepatitis B virus and human immunodeficiency virus (HIV) share common transmission routes and risk factors. Within the HIV-positive population, the global prevalence of coinfection with HBV is estimated at 7.6% (Interquartile range—IQR 5.6–12.1%) [1]. Approximately 12% of the global HIV-HBV coinfection burden is concentrated in Southeast Asia. Currently, this region ranks second only to sub-Saharan Africa in terms of overall disease impact. The prevalence of HBV infection among people living with HIV varies significantly by Asian sub-region. It ranges from a low of 5.3% in the Western Pacific to 8.8% in South and Southeast Asia, peaking at 9.4% in East Asia [1]. In Vietnam, 9.6% HBV infection has been reported among PLWH [2].

The epidemiology and clinical course of HBV are more complicated in the presence of HIV coinfection. HIV significantly reshapes the natural history of hepatitis B. It accelerates liver disease progression and disrupts the host’s immune response to HBV. In this context, conventional serological markers alone often fail to fully capture the spectrum of HBV infection. This may lead to underestimation of the true burden of HBV infection [3,4,5]. A growing concern in the management of HIV/HBV coinfection is the phenomenon of sero-reversion or even immune erasure. In such scenarios, anti-hepatitis B surface antibodies, acquired either through vaccination or past infection, wane rapidly. In result, patients become susceptible to reinfection or reactivation [6,7].

In addition, OBI, which is defined as the presence of HBV DNA in the absence of HBV surface antigen (HBsAg), is more common among PLWH. OBI can be grouped into two types based on the status of anti-hepatitis B core Ab (anti-HBc) and anti-hepatitis B surface Ab (anti-HBs). Namely, these types are “seropositive OBI” (anti-HBc positive and/or anti-HBs positive profile); and “seronegative OBI” (anti-HBc negative and anti-HBs negative profile). The latter represent a particularly challenging diagnostic entity due to the complete absence of serological markers [8,9]. These silent infections are clinically relevant. They can contribute to ongoing liver disease, pose a risk of viral reactivation, and remain undetected by routine screening strategies that rely only on HBsAg [9,10,11]. The global prevalence of OBI among PLWH is estimated at 16.26% (95% confidence interval [CI]: 10.97–22.34) [12].

Beyond host immune status, viral genetic diversity plays a critical role in shaping HBV persistence and diagnostic escape. The high replication rate of HBV, coupled with the lack of proofreading activity of its reverse transcriptase, results in substantial genetic variability [13]. Mutations within the *S* gene, especially in the major hydrophilic region (MHR), can alter antigenicity, leading to immune escape and diagnostic failure [14,15]. Various well-described substitutions (G145R, D144A/E, P120S/T, I126A/N/S/T) have been linked to impaired antibody recognition and diagnostic failure [16,17]. Importantly, due to the overlapping structure of the HBV genome, antiviral resistance mutations in the polymerase gene, such as rtM204I/V, are often accompanied by rtL180M or rtV173L. This may simultaneously induce changes in the *S* gene, thereby linking drug resistance with immune escape phenomena. This interplay is particularly relevant in HIV/HBV coinfected individuals exposed to long-term nucleos(t)ide analogue therapy [18,19].

In addition, genotypic diversity adds another layer of complexity. In Asia, genotypes B and C predominate and have been associated with different clinical outcomes and mutational patterns [20,21,22]. Literature suggests that certain genotypes, particularly C, may be more frequently associated with occult infection and immune escape mutations, although findings remain inconsistent and region-specific [23,24].

Despite such information, there is still a lack of data integrating HBV serological profiles, molecular diagnostics, genotypic distributions, and mutational patterns in HIV-infected populations in Southeast Asia, particularly in Vietnam. Therefore, this study aimed to provide a detailed analysis of these HBV factors (serological, molecular, genetic) among HIV-infected patients. By doing so, we seek to better understand the hidden burden of HBV infection in PLWH undergoing ART in southern Vietnam.

## 2. Results

### 2.1. HBV Prevalence and Profile Distribution

Participant ages ranged from 18 to 65 years (mean 35.4 ± 10.4). Among 316 HIV-infected patients, the overall prevalence values for individual markers were: 16.1% for HBsAg (95% CI: 12.5–20.6); 41.77% for anti-HBc IgG (95% CI 36.47–47.28); and 10.44% for anti-HBs IgG (95% CI 7.53–14.30). Notably, HBV DNA was detected in 103 cases (32.59%, 95% CI: 27.66–37.95%). Table 1 summarizes HBV infection status by categorization into six clinical profiles based on the aforementioned four markers. The profiles are described in the table.

The most common status was a “susceptible profile”, accounting for 36.4% of patients, followed by “anti-HBc alone” at 21.5%. CHB also showed significant prevalence at 16.1%. Notably, two cases were “HBsAg-positive yet HBV DNA-negative” within the CHB group; this may be attributable to ongoing ART. OBI was also highly prevalent, affecting 17.4% of the group. Among these OBI cases, 31 (9.8%) presented with an isolated anti-HBc marker, two had isolated anti-HBs, and one had both anti-HBc and anti-HBs. Notably, 21 patients (6.6%) had seronegative OBI, with detectable HBV DNA despite the absence of all conventional HBV serological markers. In contrast, the “presumed vaccine-immune” profile was low at 8.5%, while the “resolved infection” profile was remarkably rare at just 0.3%.

### 2.2. HBV Profiles Association with Gender and Age

Gender-based analysis showed significant differences in CHB and susceptibility (Table 2). Males had significantly higher odds of CHB than females (21.6% vs. 9.7%, aOR = 2.58, *p* = 0.005). Conversely, males were statistically less likely to be susceptible (44.1% vs. 29.8%, aOR = 0.54, *p* = 0.009).

**Figure 1:**
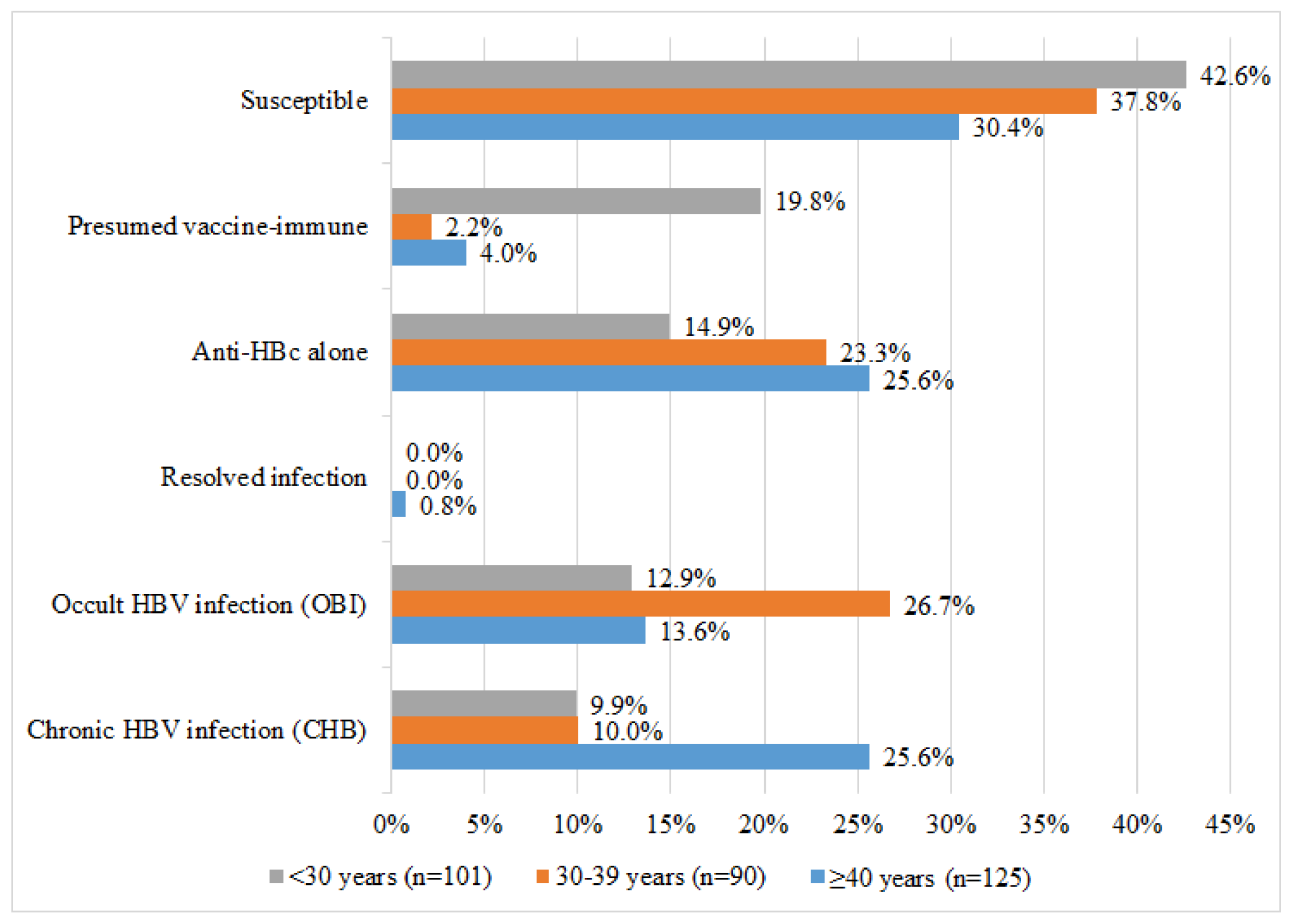
Distribution of HBV profiles by age interval.

The distribution of HBV profiles across three age groups (<30, 30–39, ≥40 years old) is shown in Figure 1. Among participants under 30, the two profiles “susceptible” and “presumed vaccine immune” were highest at 42.6% (95% CI: 33.2–52.5) and 19.8% (95% CI: 13.1–28.8), respectively. In contrast, the 30–39 age group showed markedly higher OBI (26.7%, 95% CI: 18.5–36.9). For those aged 40 and older, the data reveals a different trend: both CHB and “anti-HBc alone” reached their highest prevalence, each accounting for 25.6% (95% CI: 18.7–34.0). Interestingly, “resolved infection” remained in only a single case identified in the oldest category.

After adjusting for gender, the age-trend models revealed several distinct patterns across HBV profiles. Advancing age was strongly associated with higher prevalence of CHB (aOR = 1.87, 95% CI 1.25–2.79, *p* = 0.002). In contrast, “presumed vaccine-immune” statistically declined with age (aOR = 0.34, 95% CI 0.19–0.61, *p* < 0.001). No significant age-related trends were observed for OBI, “anti-HBc alone”, or susceptibility.

### 2.3. HBV Genotype and Sub-Genotype Distribution

The *Pre-S1/Pre-S2/S* region was successfully amplified and sequenced in all 103 HBV DNA-positive samples, which were subsequently included in the phylogenetic and mutation analyses.

**Figure 2:**
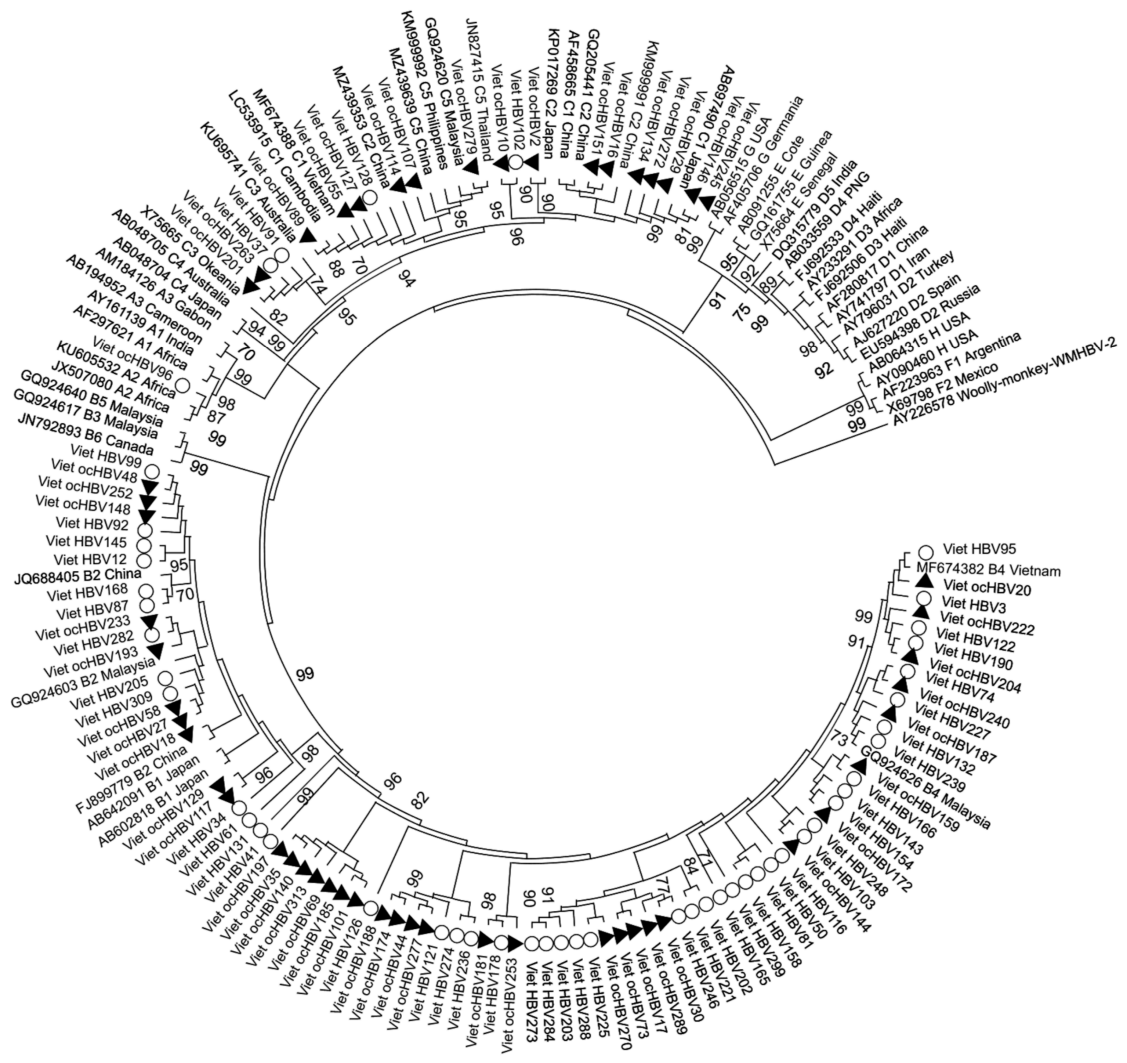
Maximum-likelihood phylogenetic analysis of HBV nucleotide sequences from HIV-infected patients. Study isolates: ○ = HBsAg+, ▲ = HBsAg−. Reference sequences from GenBank are labeled by accession number, genotype, and origin. The tree is rooted with AY226578 using bootstrap support ≥ 70 (1000 replicates).

Figure 2 shows the phylogenetic tree from the maximum-likelihood model. Our data show that genotype B was predominant (78.4%, 95% CI: 69.19–85.96), followed by genotype C (20.6%, 95% CI: 13.22–29.73) and genotype A (1.0%). At the sub-genotype level, B4 was dominant (54.9%, 95% CI: 44.74–64.78), followed by B2 (20.6%) and B3 (2.9%). Within genotype C, C1 was most common (10.8%), followed by C2 (5.9%) and C3 (3.9%). Sub-genotype A2 was rare (1.0%), and C5 was not detected. HBV genotype and sub-genotype distributions did not differ significantly by sex or age (all *p* > 0.05).

**Figure 3:**
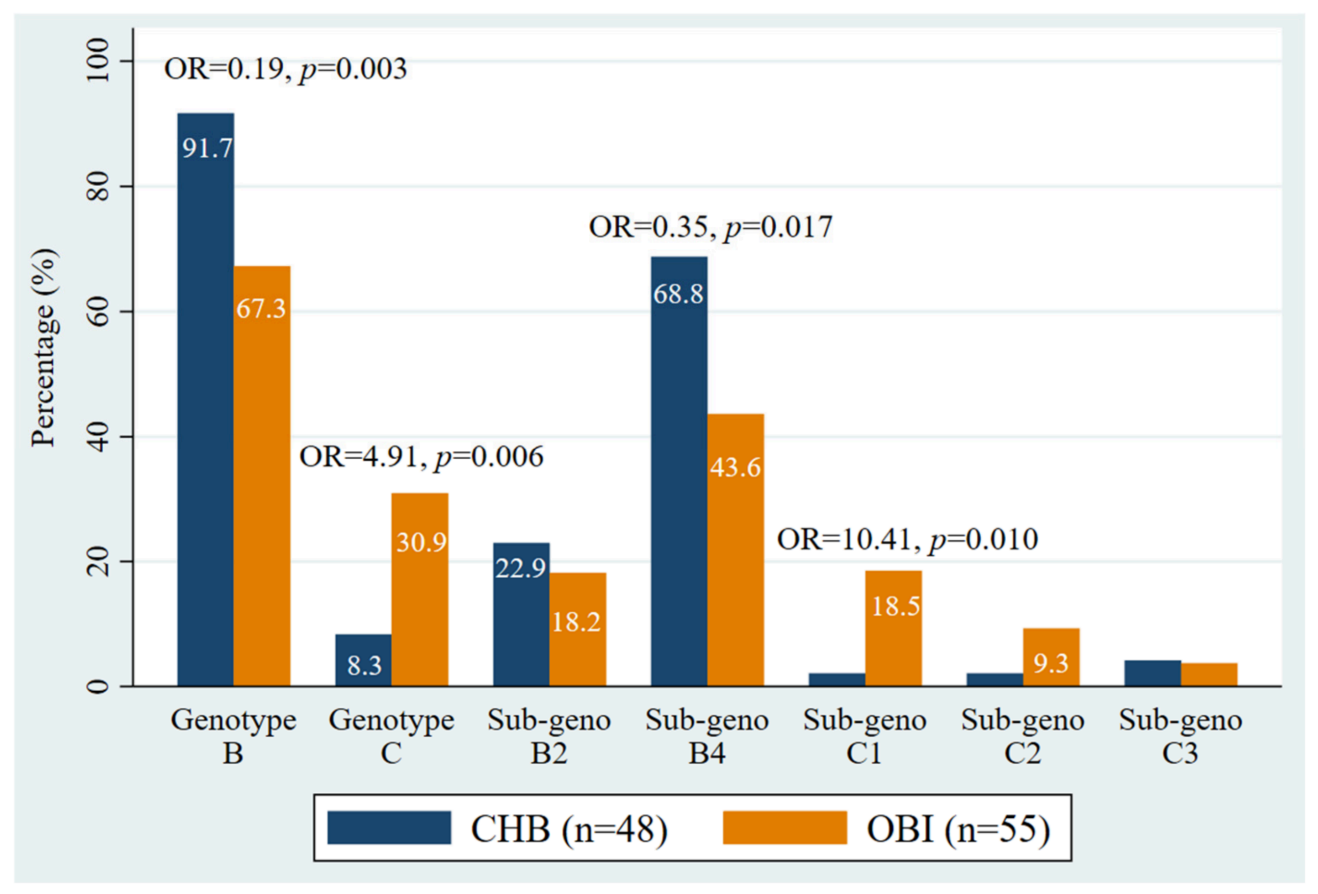
Distribution of HBV genotypes and sub-genotypes among CHB and OBI. Percentages were calculated using the total number of sequenced CHB (n = 48) or OBI (n = 55) cases as the denominator.

Figure 3 presents the distribution of genotypes and sub-genotypes among HIV/HBV coinfected patients, comparing OBI and CHB. By using Fisher’s exact test for comparison analysis, our data revealed several significant statistical variations. At the genotype level, B was highly predominant in the CHB group (91.7%, 95% CI: 80.0–97.7), but significantly lower in the OBI group (66.7%, 95% CI: 53.3–79.3, *p* = 0.003). In contrast, genotype C was significantly more frequent among OBI than CHB patients (31.5% vs. 8.3%, *p* = 0.008). This pattern was also reflected at the sub-genotype level. Sub-genotype B4, which dominated the CHB group (68.8%, 95% CI: 53.8–81.3), showed significantly lower prevalence in the OBI group (43.6%, 95% CI: 30.3–57.7, *p* = 0.013). C1 was substantially higher in OBI: 2.08% in the CHB group, yet 18.52% in the OBI group (*p* = 0.009). Other sub-genotypes did not differ between the two groups (all *p* > 0.05).

Sequence analysis of *Pre-S1* and *Pre-S2* showed that most high-frequency variants likely represent genotype-specific polymorphisms, rather than pathogenic mutations. Classical occult HBV-associated mutations such as *pre-S* deletions were not observed. Therefore, our analyses focused on the *S* gene and the overlapping *S* with reverse transcriptase (*S/RT*) domain to assess whether selection acting on *RT* substitutions could inadvertently drive mutations in *S*.

### 2.4. Mutations and Drug Resistance in the Overlapping RT/S Region

Table 3 summarizes the distribution of mutations in the overlapping *S/RT* region and their corresponding antiviral resistance profiles. Mutations were dominated by lamivudine (3TC)-associated substitutions, particularly rtM204I/V (9.71%). These were frequently accompanied by compensatory mutations such as rtL180M (6.80%) and rtV173L (3.88%). Together, these mutations cause cross-resistance to both 3TC and telbivudine (LdT) due to their shared antiviral target within the tyrosine-methionine-aspartic acid-aspartic acid (YMDD) motif of the HBV polymerase. Other mutations were observed, including: rtA181T (3.88%) associated with both 3TC and adefovir (ADV) resistance; rtA194T (2.91%) associated with tenofovir (TDF) resistance; and rtT184A (0.97%) associated with entecavir (ETV) resistance. In line with this pattern, resistance was highest for 3TC (12.62%) and LdT (11.65%), while remaining low for ETV and TDF. ADV was still susceptible. These findings indicate that 3TC resistance pathways predominate in this HIV/HBV coinfected group.

### 2.5. Mutations in the MHR Region

Many amino acid substitutions in the HBsAg MHR (aa 99–169) were detected. Among the 103 isolates analyzed, the K122R/G substitution was the most prevalent (58.3%). Other variations included M133A/F/I/L/T/V (18.5%), S143T/M/A/W (12.6%), I126A/N/S/T (11.65%), and Y161F/S (11.65%). All remaining mutations occurred at frequencies below 6%. Table 4 categorizes the distribution of major HBsAg escape mutants by group (CHB, OBI). Comparisons were performed using two-sided Fisher’s exact tests. Odds ratios were estimated with the Haldane–Anscombe 0.5 continuity correction.

Several mutations were significantly accumulated in the OBI group, including P120S/T, C124W/Y, I126A/N/S/T, and Q129H (all *p* < 0.05). In contrast, K122R was more common in the CHB group (*p* = 0.048). Other mutations, such as Y100C and M133A/F/I/L/T/V, showed higher prevalence in the OBI group but did not reach statistical significance. No differences were observed for M103I, T116N, P127T, D144A/E, C139W, S143T/M/A/W, or G145R (all *p* > 0.05).

Using the Wilcoxon rank-sum test, our data revealed that OBI carried a significantly higher number of escape mutations per patient than CHB (z = −2.26, exact *p* = 0.023). Rank sums were lower than expected in CHB (observed 2170 vs. expected 2496) and higher than expected in OBI (3186 vs. 2860). These findings indicate an increased accumulation of escape-associated mutations among patients with OBI.

To evaluate the contribution of HBsAg mutations to HBsAg seronegativity, the LASSO model was applied, with OBI status as the outcome variable. The curve plots the model’s ability to correctly identify true HBsAg-negative samples (sensitivity, *y*-axis) against its rate of incorrectly classifying true HBsAg-positive samples as negative (false positive rate, *x*-axis). The model demonstrated moderate discriminatory ability, with an area under the ROC curve of 0.76 (95% CI: 0.68–0.85). This indicates a limited, but measurable, capacity to distinguish OBI from CHB based on mutational profiles.

**Figure 4:**
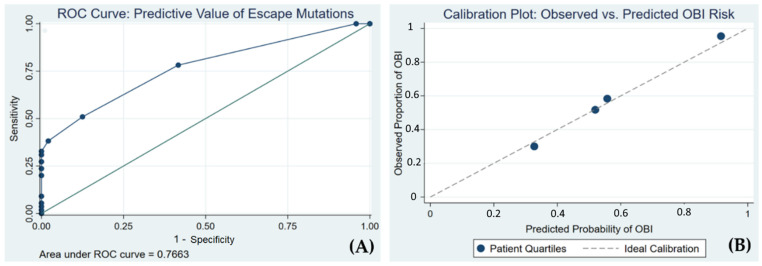
ROC and calibration plots of the LASSO model for OBI prediction in the study group. (**A**) ROC curve. The *x*-axis shows “1 − Specificity” (proportion of misclassifying HBsAg-positive samples). The *y*-axis shows “Sensitivity” (proportion of correctly identifying HBsAg-negative samples). Each dot corresponds to a different probability threshold of the model. The 45° diagonal represents random guessing (AUC = 0.5). Curves that bow farther toward the top-left corner indicate better discrimination. (**B**) Calibration plot. Patients were categorized into quartiles based on their predicted risk scores (blue circles). The dashed 45-degree line represents the ideal calibration wherein predicted probabilities perfectly match observed frequencies.

The model’s reliability was further assessed through a calibration analysis. The calibration plot showed that the predicted probabilities of OBI were in good agreement with the observed proportions across patient quartiles, as indicated by the proximity of the data points to the ideal 45-degree reference line (Figure 4). These findings suggest that although the mutational profile alone may not provide a definitive clinical diagnosis as observed among the supposedly healthy group (non-HIV), it carries relevant predictive information regarding occult HBV status.

The burden of HBsAg escape mutations did not differ by gender (*p* = 0.79) or age group (*p* = 0.67). In contrast, genotype-specific differences were observed, with C124W/Y and I126A/N/S/T substitutions occurring more frequently in genotype C than genotype B (C124W/Y substitution 23.81% vs. 1.24%, OR = 25.00, *p* = 0.001; I126A/N/S/T substitution 42.86% vs. 0%, OR = 123.88, *p* < 0.001).

## 3. Discussion

Managing HBV in PLWH remains a clinical concern globally. It is especially important in high-endemic regions like Vietnam, where screening for HBV infection is primarily based on HBsAg detection. By combining highly sensitive serological assays with molecular detection, our study describes a complex situation in this HIV-infected population. The findings suggest that the burden is not only considerable; it may also be partially hidden if current standard screening strategies are applied.

One of the most important observations is the clear gap between HBV DNA detection and HBsAg positivity. Although HBV DNA was detected in 32.6% of participants, only 16.1% tested positive for the surface antigen HBsAg. This gap underscores the high prevalence of OBI, which was identified in 17.4% of participants. These results are similar to our previous findings among apparently healthy adults in southern Vietnam. Using the same methodology in that group, we observed a similar discrepancy between DNA positivity (26.95%) and HBsAg detection (17.63%), although the OBI rate was lower at 9.3% [25]. The prevalence of HBsAg positivity observed in our study is somewhat higher than the pooled estimate of 12.11% reported in a meta-analysis of eight studies among Vietnamese PLWH conducted between 1998 and 2019 [26]. To our knowledge, local data regarding OBI in HIV-infected individuals remains quite limited. However, our findings are similar to those from a global systematic review that reported a 16.26% OBI rate (95% CI: 10.97–22.34) in HIV patients [12]. However, it is significantly higher than the 9% (95% CI: 3.0–22.0) reported in a review of Asian HIV-positive populations [27].

These differences likely reflect, at least in part, variations in assay sensitivity. To ensure the highest diagnostic accuracy, we employed highly sensitive methods in terms of limit of detection (LOD). Specifically, an HBsAg test (LOD 0.01 IU/mL) and an in-house real-time PCR HBV DNA test (5 IU/mL) were used. While many existing reviews fail to specify their detection limits, both the World Health Organization (WHO) and the Taormina Workshop on OBI recommended a sensitivity of at least 0.05 IU/mL for HBsAg and 10–20 IU/mL for HBV DNA. Notably, our assays exceed these international benchmarks, allowing us to uncover low-level infections that standard screening strategies often miss [9,28]. The clinical relevance of detecting such low-level viremia is underscored by experimental data defining the infectious dose of HBV.

It has been shown that as few as 16 viral copies (approximately 3.5 IU/mL) can establish infection. Therefore, even in patients with HBV DNA levels close to the limit of detection, the potential for viral reactivation upon immunosuppression or horizontal transmission remains non-negligible. This is particularly concerning in HIV-infected individuals who may undergo changes in immune status or antiretroviral regimen [29]. Because so many diagnostic kits are now utilized, it is vital to maintain a strict correlation between test results for making accurate clinical decisions. Standard HBsAg assays often miss low-level infections due to limited sensitivity. In fact, high-sensitivity tests can identify HBsAg in nearly half of the samples that standard assays label as negative [30,31].

In the clinical management of HIV/HBV coinfection, seronegative OBI represents an especially complicated diagnostic concern. In our study, there was a subset of seronegative OBI cases (6.6%) among overall studied HIV participants. This means seronegative OBI patients represent nearly 38% of all OBI cases in the cohort. This proportion is much higher than the standard Taormina estimates (1–20%) [9], and also exceeds that level among healthy individuals (0.5%) reported in our previous study [25]. It may reflect how heavily HIV coinfection skews the traditional epidemiological distribution of occult hepatitis.

The complete absence of serological markers in a subset of OBI cases has been reported to be silent HBV infection and raises the question of where the virus persists. Seronegative OBI has been attributed to two non-mutually exclusive mechanisms: progressive loss of HBV-specific antibodies following previous infection or failure to mount a detectable humoral response after HBV exposure [32]. In some cases, persistent HBV DNA in extrahepatic reservoirs, particularly in peripheral blood mononuclear cells (PBMCs), may further contribute to the maintenance of occult infection despite the absence of conventional serological markers [33]. In our cohort, the high proportion of seronegative OBI may reflect the combined effects of HIV-associated immune dysfunction and persistent low-level HBV infection.

The previous literature indicates that OBI is more frequent among PLWH due to immune dysfunction and impaired antibody responses [3,9,34,35]. Experts propose two primary mechanisms for seronegative OBI. Patients either progressively lose their HBV-specific antibodies over time, or they never develop an antibody response to the initial infection in the first place. In HIV care, it becomes a clinical challenge. Because the virus severely compromises adaptive immunity, it likely drives a rapid ‘immune erasure’ that extinguishes pre-existing antibodies. Consequently, the initial humoral response may be left entirely incapacitated [9]. Unfortunately, detailed immunological data, such as CD4+ T-cell counts, were not available in our study. However, it is important to note that this cohort consisted of HIV-infected individuals receiving ART who showed evidence of treatment failure, as previously reported in our study on HIV drug resistance [36]. It is reasonable to assume that a substantial degree of immune impairment may have contributed to this phenomenon.

In addition, some OBI cases may result from HBV genetic variants with *S* gene mutations, leading to undetectable modified HBsAg, despite high serum HBV DNA levels [37]. In resource-limited settings, anti-HBc is widely recognized as a practical surrogate marker for identifying potential OBI. However, our findings suggest that for HIV-infected people, the absence of anti-HBc fails to rule out the possibility of infection. If we had applied a standard screening algorithm based only on HBsAg and anti-HBc, this 6.6% of patients would have been misclassified as simply “susceptible” to HBV. Consequently, they would be denied essential monitoring for liver disease progression. They would also remain unprotected against the severe risk of viral reactivation if their immunosuppression worsened.

Interestingly, our study found that genotype C was significantly more frequent in the OBI group compared to patients with CHB (31.5% vs. 8.3%, *p* = 0.008), despite genotype B’s predominance overall. This fact is consistent with evidence that host immune status plays a dominant role in OBI development. A similar trend was evident at the sub-genotype level, with a marked increase in sub-genotype C1 among OBI cases. This finding suggests a potential advantage of genotype C variants in maintaining occult persistence under HIV-related immune deficiency [9,38]. This is especially concerning because genotype C is already notorious for causing more aggressive liver disease and a higher risk of HCC in Asia [3,39,40,41].

Beyond genotype, specific mutations in the basal core promoter (BCP) and precore regions, such as the double mutation A1762T/G1764A and the G1896A (W28 stop) substitution, have been independently associated with an increased risk of liver cirrhosis and hepatocellular carcinoma (HCC), even in patients with low or undetectable viral loads. These mutations can be detected years before HCC onset and may serve as early markers of hepatocarcinogenesis [42,43,44]. In the context of HIV/HBV coinfection, where liver disease progression is already accelerated, the presence of such mutations in patients with occult infection warrants closer monitoring, even when standard liver function tests appear normal.

Furthermore, the “resolved HBV infection” profile was almost absent in our study, accounting for 0.3% of the overall study group. This contrasts sharply with the higher value found in the healthy population in our previous study, where resolved infection reached 14.1% [25]. Our findings are closely aligned with a recent 13-year longitudinal study by Kinai et al., where not a single participant exhibited a true resolved infection pattern. Their research confirms a critical point: anti-HBc alone and the complete loss of HBV antibodies in PLWH do not signify that the infection has been resolved. Instead, such profiles point to a fragile and unstable immune state [45].

We observed a similar trend. Our data suggests that HIV/HBV coinfected patients might transition into categories associated with increased vulnerability. Indeed, the distribution of serological profiles in our study participants supports this interpretation. The largest proportion of the cohort (36.4%) exhibited a “susceptible profile”, while another 21.5% had the “anti-HBc alone profile”. At first glance, the “susceptible profile” may suggest individuals who have never been exposed to HBV. However, in the context of HIV infection, this interpretation may be overly simplistic. It is also plausible that a subset of these individuals had prior exposure, yet as a result of progressive immune dysfunction lost detectable antibodies or even featured transiently undetectable HBV viral DNA. This phenomenon is consistent with the concept of immune erasure, whereby HIV-related impairment leads to the gradual loss of serological memory [38,46]. The high proportion of susceptible individuals underscores the importance of routine HBV serological screening and timely vaccination of PLWH without evidence of protective immunity, as recommended by current international guidelines. Individuals with an isolated anti-HBc profile may also require further evaluation, including HBV DNA testing when clinically indicated, as this profile can represent OBI rather than resolved infection alone [47,48].

Further complexity is highlighted by the observation that two patients in our cohort were HBsAg-positive yet HBV DNA-negative. This discordance may reflect the suppressive effect of ART, particularly regimens containing HBV-active agents, such as TDF or 3TC. These can reduce viral replication to levels below the threshold of detection, yet HBsAg remains detectable. Alternatively, it may represent fluctuating viral dynamics or low-level replication not captured at a single time point. Taken together, these findings underscore the dynamic, and often unstable, nature of HBV infection in PLWH, where serological and virological markers may not consistently align. Overall, this near-zero rate of viral resolution clearly highlights how poorly the compromised immune systems of HIV patients manage to naturally clear and control the hepatitis B virus over time.

When analyzing amino acid substitutions in the *S* gene MHR region, it appeared that HBsAg escape mutations were a molecular predictor driving OBI. These mutations seemed to play a central role in OBI, stemming from the interaction between viral variability and host immunity. The mechanistic basis for diagnostic failure in the presence of these mutations involves alterations in the hydrophilicity, electrical charge, and/or acidity of the HBsAg protein, particularly within the MHR. Such conformational changes reduce the binding affinity of HBsAg for both diagnostic antibodies and vaccine-induced neutralizing antibodies [49,50]. In our cohort of PLWH, the burden of such mutations was clearly higher in OBI than in CHB. Using the Wilcoxon rank-sum test, we found that OBI cases carried a significantly greater number of escape mutations per patient (z = −2.26, exact *p* = 0.023). This observation is consistent with prior reports indicating that OBI is frequently associated with the accumulation of surface gene mutations that impair HBsAg secretion or recognition [9,38]. In other words, the virus becomes less visible, both to the immune system and to standard diagnostic assays.

Notably, similar escape mutations have been documented in circulating HBV variants from other geographic regions and population groups, including pregnant women in Europe. This reinforces that this phenomenon is not limited to HIV-endemic settings, but rather represents a general challenge for HBV serological screening [51]. Several substitutions (including P120S/T, C124W/Y, I126A/N/S/T, Q129H) were more accumulated in OBI than CHB, with some mutations showing association with genotype C. This is not surprising. Prior studies have suggested that genotype C may have a greater tendency toward immune escape and persistence, possibly reflecting distinct evolutionary dynamics under sustained immune pressure [23,24,40].

To further explore the predictive value of these mutational patterns, we applied LASSO-penalized logistic regression. The resulting model based on *S*-gene mutations demonstrated moderate discriminatory performance (AUC = 0.76). This is lower than what we previously found with conditionally healthy individuals (AUC = 0.83) [25]. Such a difference may suggest the additional influence of host-related factors in HIV-infected patients. Indeed, impaired humoral immunity and B-cell dysfunction associated with HIV infection likely weaken the direct relationship between viral mutations and serological outcomes. These dynamics make OBI a more complex and multifactorial condition in this population.

In addition, it is interesting that we also observed some major mutations in the overlapping *RT/S* region among HIV/HBV coinfected patients. Because the polymerase and surface genes overlap, resistance-associated substitutions identified in this region should be interpreted within this unique genomic organization, whereby a single nucleotide substitution has the potential to affect both reading frames simultaneously. Previous studies have demonstrated that key RT resistance mutations, particularly those selected by 3TC, may be accompanied by amino acid substitutions in the overlapping *S* gene. For example, the rtM204V/I, rtL180M, and rtA181T mutations have been shown to map to changes within or near the HBsAg determinant which contributes to immune escape or diagnostic failure [13,52,53]. In our study, 12.62% of HIV/HBV coinfected patients harbored mutations associated with 3TC resistance. These predominantly were M204I/V (9.71%) and L180M (6.80%), with additional mutations such as A181T/V and V173L. Our previous HIV drug resistance study of this cohort showed all participants received 3TC-containing ART, with a mean treatment duration of 5.45 years, and a relatively high prevalence of virological failure, as well as HIV drug resistance after prolonged ART exposure [35]. Although HIV and HBV resistance mechanisms are distinct, these findings indicate sustained antiviral treatment pressure in this population and provide relevant therapeutic context for the predominance of lamivudine-associated HBV polymerase mutations observed in the present study. Unfortunately, detailed individual treatment histories, including cumulative 3TC exposure, were unavailable; therefore, we could not directly evaluate the relationship between treatment duration and resistance development. Moreover, due to a cross-sectional study, it should not be interpreted as evidence of a causal relationship between antiviral exposure, RT resistance mutations, and alterations in the S gene.

This study has several strengths. It combines highly sensitive serological and molecular assays, enabling detection of low-level markers that might otherwise be missed. In addition, the integration of genotypic and mutational analyses provides a more comprehensive understanding of the additional complication of HBV infection in HIV-infected patients. To our knowledge, this is among the first studies in Vietnam to simultaneously evaluate HBV infection profiles, OBI burden, and *S* gene mutational patterns in this population.

### Limitations of the Study

This study has several limitations. First, the cross-sectional design does not allow us to assess longitudinal changes in HBV serological markers, including potential sero-reversion or temporal fluctuations in HBV DNA levels. Second, the analysis relied primarily on demographic data extracted from medical records without detailed clinical or immunological information, such as prior HBV infection status, hepatitis B vaccination history, or concurrent CD4^+^ T-cell counts. The absence of these data limits our ability to fully interpret the relationships between host immune status, the occurrence of OBI, and the accumulation of *S* gene escape mutations. Third, the study population was recruited from selected outpatient clinics in southern Vietnam. This may somewhat limit the generalizability of our findings to other regions or to HIV-infected populations with different ART coverage or baseline immune characteristics. Despite these constraints, the study provides important insights into the hidden burden of HBV infection and the molecular basis of OBI in this high-risk population, while also highlighting areas for future research.

## 4. Materials and Methods

### 4.1. Materials

Our study enrolled 316 HIV-infected patients on ART in various out-patient-clinics (OPCs) in southern Vietnam. We collected 10 mL of whole blood per patient during routine clinical monitoring for ART. As described previously for this cohort [36], at enrollment all participants were receiving 3TC-containing ART as the Vietnam national HIV treatment guideline, with a mean treatment duration of 5.45 years. Approximately half of the cohort also received TDF as part of combination ART. The research was conducted under ethics approvals obtained from the Saint Petersburg Pasteur Institute Ethics Committee (protocol No. 47, 25 December 2018; protocol No. 92, 29 October 2019), and under a Memorandum of Understanding signed 15 December 2017 between the Saint Petersburg Pasteur Institute (Russia) and the Pasteur Institute in Ho Chi Minh City (Vietnam). After receiving a thorough briefing on the study’s objectives, participants provided their written informed consent. Demographic data only were extracted from de-identified medical records to ensure strict patient confidentiality.

Samples were processed as described previously in our earlier study [25]. Briefly, plasma was isolated by centrifugation (1500× *g*, 20 min, 4 °C), followed by ultracentrifugation (24,000× *g*, 1 h) to enrich viral particles. Concentrated plasma samples were aliquoted for downstream analyses, including serological testing, molecular screening, and sequencing applications.

### 4.2. HBV Testing

#### 4.2.1. Serological and Molecular Biological Assays

All serological and molecular biological assays were performed as previously described in our earlier study [25]. Briefly, three HBV serological markers were screened (HBsAg, anti-HBs IgG, anti-HBc IgG) using qualitative enzyme-linked immunosorbent assays (ELISA) [25]. To ensure technical precision, all samples were tested in duplicate following manufacturers’ instructions. The study utilized commercial kits from Diagnostic Systems RPC (Nizhny Novgorod, Russia) and Vector-Best (Novosibirsk, Russia). Notably, the assay used for HBsAg detection featured high analytical sensitivity, with a LOD of 0.01 IU/mL.

For molecular analysis, total nucleic acids were extracted from blood plasma using the Ribo-Prep commercial kit (CRIE, Moscow, Russia). Primary HBV DNA detection was then conducted via real-time PCR using the AmpliSens HBV-FL kit (CRIE, Moscow, Russia), which has an LOD of 50 IU/mL. To capture cases of low-level viremia, samples were further analyzed using a highly sensitive in-house PCR method developed by the Saint Petersburg Pasteur Institute, achieving a superior LOD of 5 IU/mL [25,54,55,56]. We applied a stepwise extraction and amplification protocol based on sample volume: 200 μL for initial screening; a blinded repeat from a fresh 200 μL aliquot to confirm all positive results; 500 μL for inconclusive PCR cases; and ≥5 mL for sequencing. Each run included a negative control (sterile PBS) [57].

#### 4.2.2. HBV Genotype and Mutation Analysis

For all samples confirmed to be HBV DNA-positive, the *Pre-S1*, *Pre-S2*, and *S* regions were then amplified for sequencing using a nested PCR protocol with a set of flanking primers [9,18,19]. After sequencing, all raw fragment data were analyzed using Unipro UGENE (v.51). These fasta sequences were initially identified via the BLASTn algorithm (https://blast.ncbi.nlm.nih.gov/) against the GenBank database and subsequently deposited therein. The nucleotide sequences obtained in this study have been deposited in the GenBank database under accession numbers MN535017–MN535019, MT740718–MT740720, MZ675785–MZ675807, PX475816–PX475833, and PZ244443–PZ244498. For phylogenetic analysis, sequences were aligned using the ClustalW algorithm in MEGA12. This analysis applied the Tamura-Nei genetic distance model and the maximum-likelihood tree-building method with a 1000 bootstrap value. Sequences were also screened for mutations using the HBVseq, HBVdb, and Geno2pheno online databases [58,59,60].

### 4.3. Statistical Analysis

Data were analyzed using STATA version 17 (StataCorp LLC, College Station, TX, USA). Categorical variables were summarized as frequencies and percentages, with 95% CIs calculated using the exact (Clopper–Pearson) method. Differences in proportions were assessed using Pearson’s chi-square test or Fisher’s exact test when expected cell counts were below 5, with the Haldane–Anscombe correction applied for zero-cell counts. Continuous variables were compared using the Wilcoxon rank-sum (Mann–Whitney) test. A two-sided *p*-value < 0.05 was considered statistically significant.

To evaluate the predictive power of escape mutations for OBI, a penalized logistic regression model with a least absolute shrinkage and selection operator (LASSO) was developed. Its performance was assessed by the AUC. AUC values were interpreted as follows: values <0.6 indicate poor or no discrimination; 0.6–0.7 weak; 0.7–0.8 moderate; 0.8–0.9 good; and >0.9 excellent discriminatory ability [61,62].

## 5. Conclusions

In conclusion, our findings show that HBV infection among HIV-infected individuals in southern Vietnam is not only common, but also complex. Both CHB and OBI are highly prevalent, with a high proportion of seronegative cases. At the molecular level, the accumulation of *S* gene escape mutations, together with the higher frequency of genotype C in OBI, points to a dynamic interplay between viral evolution and host immune pressure. Taken together, our results underscore the limitations of conventional screening strategies that rely solely on standard serological markers. They also highlight the need for a more integrated diagnostic approach. In high-risk populations such as PLWH, combining highly sensitive serological assays with molecular techniques may be essential for accurate detection and appropriate clinical management.

## Figures and Tables

**Table 1:** Distribution of HBV serological and molecular profiles.

	HBsAg	Anti-HBs IgG	Anti-HBc IgG	HBV DNA	n	Percentage (95% CI)
1. Chronic HBV infection (CHB)	(+)	(+/−)	(+)	(+/−)	50	15.8% (11.98–20.3)
2. Occult HBV infection (OBI)	(−)	(+/−)	(+/−)	(+)	55	17.4% (13.4–22.0)
3. Resolved HBV infection	(−)	(+)	(+)	(−)	1	0.3% (0.0–2.2)
4. Anti-HBc alone	(−)	(−)	(+)	(−)	68	21.5% (17.3–26.4)
5. Presumed vaccine immune	(−)	(+)	(−)	(−)	27	8.5% (5.9–12.2)
6. Susceptible	(−)	(−)	(−)	(−)	115	36.4% (31.3–41.9)

**Table 2:** Gender-based comparison of HBV profiles with frequency (n) and odds ratios.

	Male		Female		Fisher’s Test	
	n	% (95% CI)	n	% (95% CI)	OR (95% CI)	*p*-Value
Chronic HBV (CHB)	37	21.6% (16.1–28.5)	14	9.7% (5.8–15.7)	**2.58 (1.33–5.00)**	**0.005**
Occult HBV (OBI)	31	18.1% (13.0–24.7)	23	15.9% (10.7–22.8)	1.17 (0.65–2.12)	0.594
Resolved infection	0		1	0.7% (0.1–4.8)		
Anti-HBc alone	42	24.6% (18.7–31.6)	26	17.9% (12.5–25.1)	1.49 (0.86–2.58)	0.154
Presumed vaccine immune	10	5.8% (3.2–10.6)	17	11.7% (7.4–18.1)	0.47 (0.21–1.06)	0.068
Susceptible	51	29.8% (23.4–37.2)	64	44.1% (36.2–52.4)	**0.54 (0.34–0.86)**	**0.009**

Note: Bold entries indicate significant values.

**Table 3:** Mutations in the overlapping *S/RT* region associated with antiviral resistance among HIV/HBV coinfected patients.

Interpretation		3TC	ADV	ETV	LdT	TDF
Resistant (%)		12.62	0	1.94	11.65	0.97
Possible resistance (%)		0.97	0	6.8	0	0
Susceptible (%)		86.41	100	91.26	88.35	99.03
**Mutation**	**%**					
rtM204I/V	9.71	X		X	X	
rtL180M	6.80	X			X	
rtA181PT	3.88	X	X		X	
rtV173L	3.88	X				
rtA194T	2.91					X
rtM204S	1.94	X				
rtT184A	0.97			X		

**Table 4:** Distribution of escape mutations among HIV/HBV coinfected patients with CHB and OBI.

Mutation	Occurrence in CHB Group (N = 48)		Occurrence in OBI Group (N = 55)		OBI vs. CHB (Fisher’s Exact)	
	n	%, 95% CI	n	%, 95% CI	OR	*p*-Value
Y100C	0	0%	5	9.09 (3.02–19.95)	10.56	0.059
M103I	1	2.08 (1.05–11.07)	3	5.45 (1.14–15.12)	2.11	0.621
T116N	0	0%	1	1.82 (0.05–9.72)	2.67	1.000
P120S/T	0	0%	6	10.91 (4.11–22.25)	12.74	0.029
K122R	33	68.75 (33.75–81.34)	27	49.09 (35.35–62.93)	0.45	0.048
C124W/Y	0	0%	6	10.91 (4.11–22.25)	12.74	0.029
I126A/N/S/T	1	2.08 (1.05–11.07)	11	20.00 (10.43–32.97)	8.18	0.005
P127T	2	4.17 (2.51–14.25)	0	0%	0.17	0.215
Q129H	0	0%	6	10.91 (4.11–22.25)	12.74	0.029
M133A/F/I/L/T/V	5	10.42 (5.47–22.66)	14	25.45 (14.67–39.00)	2.76	0.074
C139W	1	2.08 (1.05–11.07)	0	0%	0.29	0.466
S143T/M/A/W	6	12.50 (6.73–25.25)	7	12.73 (5.27–24.48)	1.01	1.000
D144A/E	0	0%	5	9.09 (3.02–19.95)	10.56	0.059
G145R	0	0%	2	3.64 (0.44–12.53)	4.53	0.497

## Data Availability

The original contributions presented in this study are included in the article. Further inquiries can be directed to the corresponding author.
